# Self-adjuvanting C18 lipid vinil sulfone-PP2A vaccine: study of the induced immunomodulation against *Trichuris muris* infection

**DOI:** 10.1098/rsob.170031

**Published:** 2017-04-12

**Authors:** M. Gomez-Samblas, J. J. García-Rodríguez, M. Trelis, D. Bernal, F. J. Lopez-Jaramillo, F. Santoyo-Gonzalez, S. Vilchez, A. M. Espino, F. Bolás-Fernández, A. Osuna

**Affiliations:** 1 Instituto de Biotecnología, Grupo de Bioquímica y Parasitología Molecular, Departamento de Parasitología, Universidad de Granada, Campus Universitario Fuentenueva, 18071 Granada, Spain; 2 Instituto de Biotecnología, Grupo de Bioquímica y Parasitología Molecular, Departamento de Bioquímica, Universidad de Granada, Campus Universitario Fuentenueva, 18071 Granada, Spain; 3 Departamento de Parasitología, Facultad de Farmacia, Universidad Complutense, Plaza de Ramón y Cajal s/n. Ciudad Universitaria, 28040 Madrid, Spain; 4 Àrea de Parasitologia, Departament de Farmàcia i Tecnologia Farmacèutica i Parasitologia, Universitat de València, Av. V.A. Estellés, s/n, 46100 Burjassot (Valencia), Spain; 5 Joint Research Unit on Endocrinology, Nutrition and Clinical Dietetics, Health Research Institute-La Fe, Universitat de Valencia, Av. Fdo. Abril Martorell, 106, 46026 Valencia, Spain; 6 Departament de Bioquímica i Biologia Molecular, Universitat de València, C/ Dr Moliner, 50, 46100 Burjassot (Valencia), Spain; 7 Departamento de Química Orgánica, Facultad de Ciencias, Instituto de Biotecnología, Universidad de Granada, 18071 Granada, Spain; 8 Laboratory of Immunology and Molecular Parasitology, Department of Microbiology, University of Puerto Rico, School of Medicine. PO Box 365067, San Juan 00936-5067, Puerto Rico.

**Keywords:** *Trichuris muris* vaccination, self-assembling lipopeptide rPP2A, chemokines, cytokines, lipid vinyl sulfone

## Abstract

Despite the importance of the adjuvant in the immunization process, very few adjuvants merge with the antigens in vaccines. A synthetic self-adjuvant oleic-vinyl sulfone (OVS) linked to the catalytic region of recombinant serine/threonine phosphatase 2A from the nematode *Angiostrongylus costaricensis* (rPP2A) was used for intranasal immunization in mice previously infected with *Trichuris muris*. The animal intranasal immunization with rPP2A-OVS showed a reduction of 99.01% in the number of the nematode eggs and 97.90% in adult. The immunohistochemical analysis of the intestinal sections showed that in immunized animals with lipopeptide the mucus was significantly higher than in the other experimental groups. Also, these animals presented significantly different chemokine, CCL20 and CCL11, levels. However, although the number and size of Tuft cells did not vary between groups, the intensity of fluorescence per cell was significant in the group immunized with the rPP2A-OVS. The results of the present study suggest that mice immunized with the lipopeptide are capable of activating a combined Th17/Th9 response. This strategy of immunization may be of great applicability not only in immunotherapy and immunoprophylaxis to control diseases caused by nematodes but also in pathologies necessitating action at the level of the Th9 response in the intestinal mucosa.

## 1. Introduction

Helminths have plagued human beings even before recorded history. Today, it is estimated that roughly one-third of the almost 3 billion people who live on less than 2 USD per day in developing regions of sub-Saharan Africa, Asia and the Americas are infected with one or more species of helminths [1], exacerbating poverty, malnutrition and anaemia as well as causing delays in the physical and intellectual development of infants [2], the most susceptible population. The most common human helminthiases involve infection with intestinal helminths, such as ascariasis, trichuriasis and hookworms, followed by schistosomiasis. Moreover, helminth infections represent a significant economic and health burden to the global ruminant livestock industry, causing huge losses in meat and milk production. Also, resulting in a high mortality rate and the excessive use of anthelmintic treatments [3,4], their effectiveness has been compromised in many geographical areas due to growing resistance [5]. Thus, there is an urgent need to develop new measures to control helminth infections, such as vaccinations [6–8]. We have previously demonstrated that the catalytic region of the enzyme serine/threonine phosphatase 2A (PP2A), which is highly conserved among a large number of nematode species [9], is capable of inducing partial levels of protection against infections by diverse parasitic nematodes when administered by the mucosal route, specifically intranasally, in mice and lambs.

Mucosal immunity involves an intricate network of components of innate and adaptive immunity [10] that act independently of the systemic immune response [11]. In the mucosa, the antigens make contact with the local immune system both by antigen-presenting cells (APC) as well as ‘specific microfold cells’ (M) that are intercalated in the mucosa with follicles of mucosa-associated lymphoid tissue (MALT) in association with Peyer's patches (PPs). Mucosa covers both the aerodigestive and the urogenital tract, constituting a special immune system, as this mucosa is the entry pathway of almost 90% of infections in vertebrates. Despite being anatomically separate, mucosa acts synergistically; for example, intranasal immunization stimulates T or B cells to respond in the intestinal or the urogenital mucosa [12], thus being an alternative to intramuscular, intradermal or subcutaneous vaccination.

Apart from the administration route, the induction of the immune response depends on the incorporation of an appropriate adjuvant that triggers the innate response and is capable of tolerating the transition from an innate response to an adaptive response. Some reviews on the use of adjuvants in the immunization through mucosa have recently been published [12–18]. By the use of a bioconjugation methodology, a number of soluble proteins, including ovalbumin (OVA), cytochrome *c*, Tamm Horsfall glycoprotein and HIV-1 gp120, have been attached to palmitic acid via the ɛ-amine groups of lysine [19]. The first work in which lipopeptides were used as adjuvants were performed by Hopp [20]. Other authors [21,22] found that a lipopeptide of influenza triggered a cytotoxic CD8+ T lymphocyte (CTL) response. This finding enormously boosted the potential use of lipids bound to peptides as vaccines [23]. Conjugated peptides with tripalmitoyl-*S*-glycerylcysteinyl-seryl-serine (P3CSS) induced a CTL response similar to that achieved when live vaccines are used [24,25].

This study tests the ability of recombinant PP2A subunit from the parasitic nematode *Angiostrongylus costaricensis* to induce cross-protective immune responses against a challenge *Trichuris muris* infection in mice when the subunit is administered intranasally and is formulated as a lipoconjugate with oleic-vinyl sulfone (OVS). Results demonstrate that this formulation induces a strong protective immunity in the infected and vaccinated animals. The rapid expulsion of the intestinal parasites in the PP2A-OVS vaccinated animals was found to be associated with the high production of IL-9 in the PPs.

## 2. Results

### 2.1. Synthesis of the oleic-vinyl sulfone

**Figure 1.**
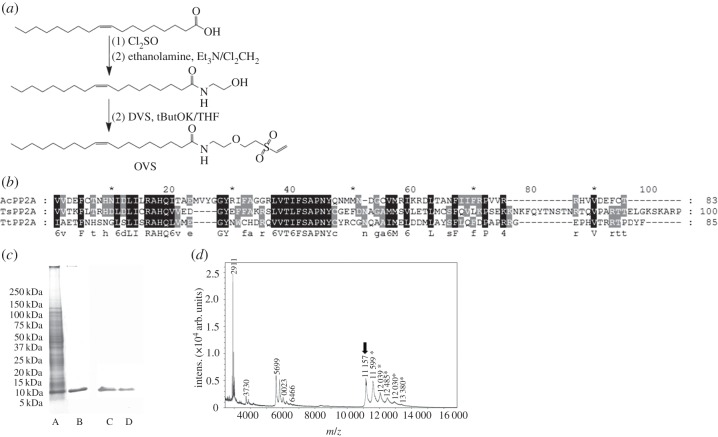
Recombinant peptide (rPP2A), oleic-vinyl sulfone (OVS) and lipopeptide (rPP2a-OVS). (*a*) The synthesis of (9Z)-*N*-[2-(ethenylsulfonyl)ethoxy]-ethyl]-9-octadecenamide (OVS). (*b*) Multiple alignments (ClustalW2) of the sequence of the catalytic region of *Angiostrongylus costaricensis* PP2A and different *Trichuris* ssp. Ac, *Angiostrongylus costaricensis*; Ts, *Trichuris suis*; Tt, *Trichuris trichiura.* Black indicates the positions with 100% conservation, while grey represents a decline in conservation. (*c*) SDS-PAGE analysis of purified PP2A and western blot. Lane A, total protein transformed bacteria; lane B, PP2A after affinity chromatography with nickel-agarose column purification; lanes C and D, recognition by the immune serum against rPP2A. (*d*) Mass spectrometry of linked rPP2A to OVS. The arrow corresponds to rPP2A, and asterisks to fusions of 1, 2, 3, 4 and 5 OVSs linked to rPP2A.

The oleic acid was transformed into the vinyl sulfone derivate as described by Morales-Sanfrutos *et al*. [26], in the three steps depicted in figure 1. Compound (9Z)-*N*-[2-(ethenylsulfonyl)ethoxy]-ethyl]-9-octadecenamide was obtained as a solid (279 mg, 81%). M. P. 61–62°C; nmax(film) cm^−1^: 3062, 2915, 2847, 1462, 1284, 1124, 908 and 730; ^1^H-NMR(CDCl_3_, 400 MHz): d 6.62 (dd, 1H, *J* = 16.6 and 9.8 Hz, CH=), 6.43 (d, 1H, *J* = 16.6 Hz, =CH_2_*trans*), 6.15 (d, 1H, *J* = 9.8 Hz, =CH_2_*cis*), 2.96 (t, 2H, *J* = 8.0 Hz), 1.77 (m, 2H), 1.46–1.20 (m, 30H), 0.88 (t, 3H, *J* = 6.7 Hz); ^13^C-NMR (CDCl_3_, 100 MHz): d 136.3, 130.4, 54.4, 32.0, 29.8, 29.7, 29.7, 29.7, 29.6, 29.4, 29.3, 29.1, 28.5, 22.8, 22.4, 14.2. HRMS (*m*/*z*) (FAB+) calcd. for C_20_H_40_O_2_SNa [M + Na]^+^: 367.2647; found: 367.2644.

### 2.2. Lipopeptide: antigen and self-assembly with the oleic-vinyl sulfone

After the sequencing and analysis of the recombinant peptide and search by Mascot, the recombinant protein was identified as a homologue of the catalytic subunit of the family serine/threonine phosphatase 2A, with a value of *E* = 2 × 10^−16^. The sequence is shown in figure 1. The analysis of the nucleotide sequence of the recombinant protein (rPP2A) using the ClustalW algorithm (http://www.ebi.ac.uk/Tools/msa/clustalw2/) confirmed the high identity between the sequence of the catalytic region of recombinant serine/threonine phosphatase 2A from *A. costaricensis* (AcPP2Ar) and the catalytic region of the PP2A from *Trichuris suis* (TsPP2A) and *T. trichiura* (TtPP2A).

The analysis of the recombinant protein by 12.5% SDS-PAGE electrophoresis showed that the molecular weight of the recombinant protein was 10 kDa, and the theoretical molecular mass was 9898.4 Da, with an isoelectric point value of pI 9.30 based on the sequence calculated by the ExPASy pI/Mw tool (http://web.expasy.org/compute_pi/). Western blot confirmed the identity of the purified protein (figure 1). The recombinant protein was chemically bounded to OVS, yielding a lipopeptide with rPP2A coupled to 1 or 2 molecules of OVS with minor amounts of 3 and 4 OVS molecules and traces of 5 OVS molecules as revealed by mass spectrometry (figure 1).

**Figure 2.**
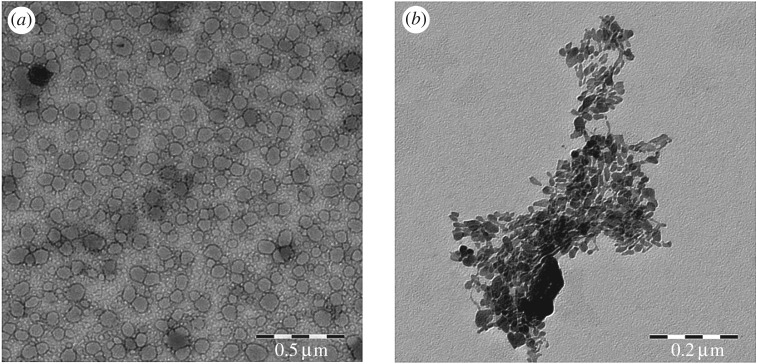
Transmission electron microscopy images. (*a*) Micelles formed after the functionalization of the rPP2A with OVS. (*b*) Purified rPP2A in aqueous suspension.

Notably, the electron microscope revealed that the lipopeptide formed micelles with 46.66 ± 6.40 nm in diameter (figure 2). Figure 2 shows that the purified rPP2A (not linked to the lipid vinyl sulfone) in a PBS suspension forms aggregates as the result of its low solubility in aqueous solution.

### 2.3. Evaluation of the anti-helminth activity

**Figure 3.**
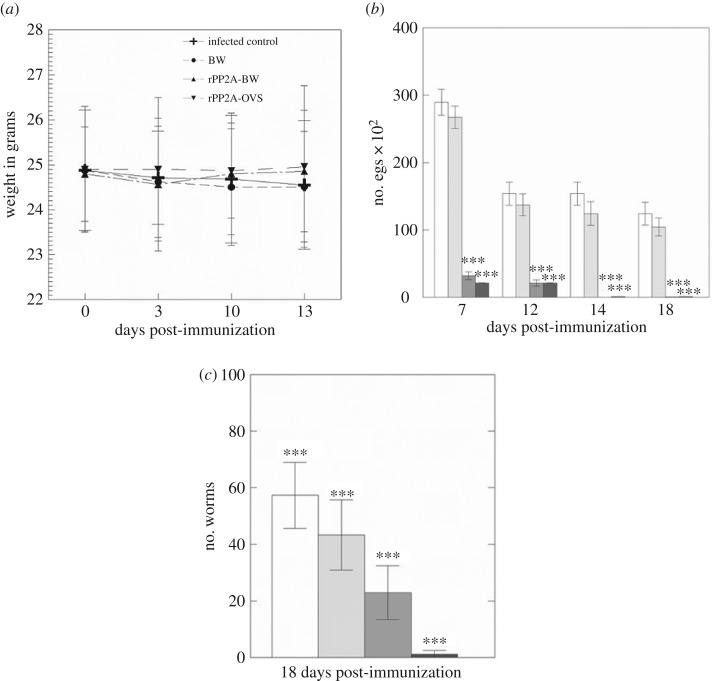
(*a*) Animal weight (average in grams) from the different immunized groups after the second immunization. No differences were obtained among the groups. (*b*) Number of eggs along time present in each animal group post-immunization. The number of eggs present in rPP2A-OVS immunized animals was significantly lower than in the control group and in those immunized with BW. (*c*) Number of worms found on average in each immunized group. The differences were statistically significant (*p* *<* 0.001, ***) in a Tukey–Kramer test. White bars show the IC group; grey bars the BW immunization; dark grey bars rPP2A-BW immunization and back bars the rPP2A-OVS immunization.

To evaluate the possible adverse effects of the different intranasal inoculation treatments that the different groups of animals underwent, we weighed the mice throughout the experiment, comparing them to the IC group (figure 3). The results indicate that there was no variation among the animals of the different groups, and the variability of the weights in the IC group was even higher than in the rest of the experimental groups. Although without statistical significance (*p* > 0.05), at the end of the experiment, the mice immunized with rPP2A-OVS and rPP2A-BW increased slightly in weight when compared with the IC, probably because of the parasite loads, as discussed below (figure 3).

The average number of eggs expelled by the different groups of mice after immunization is represented in figure 3, showing that one week post-immunization the immunized mice with the formulae that included rPP2A-BW and rPP2A-OVS reached percentages of 88.93% and 92.73% reduction of the number of eggs, respectively. Curiously, in the mice treated only with the BW at 7 days post-immunization a reduction of 7.67% was reached. At 12 days post-immunization, the groups immunized with rPP2A-BW and rPP2A-OVS reached minimum values, not being significant when compared with each other but highly significant compared with the IC and to the BW groups (Tukey–Kramer test, *p* *<* 0.001). Two weeks post-immunization the number of eggs in mice treated with BW declined 16.13% compared with IC, plunging 99.85% in those treated with rPP2A-BW and 99.01% for those treated with rPP2A-OVS. This reduction not only remained steady but persisted until day 18 of the experiment.

The number of worms in the control animals killed at 14 days post-infection (p.i), before beginning the immunization, was 83 ± 31.85, demonstrating that the infection was successful and that *T. muris* was correctly established. The fall in the number of worms compared with the control group at the end of the experiment (when the animals were killed) is shown in figure 3, indicating that the animals immunized with rPP2A-OVS had 97.90% less worms than mice immunized with PP2A-BW (59.88%), and those treated only with BW (24.47%).

### 2.4. Interleukin expression assessment by quantitative real-time PCR and evaluation of mucosal immune changes by confocal microscopy

The evaluation of the immune response of the different groups of animals carried out as described in Material and methods by laser confocal microscopy in thin sections of the intestinal caecum. The levels of fluorescent areas of the mucine zones, antibody recognition against chemokines CCL20, CCL11 and OX40, neutrophils, the plasma-cell marker CD138 and Tuft cells were determined. Also, the expression levels of different interleukins were evaluated using quantitative real-time PCR, measuring the mRNA-production levels of these proteins both in the PPs and in the MLN.

**Figure 4.**
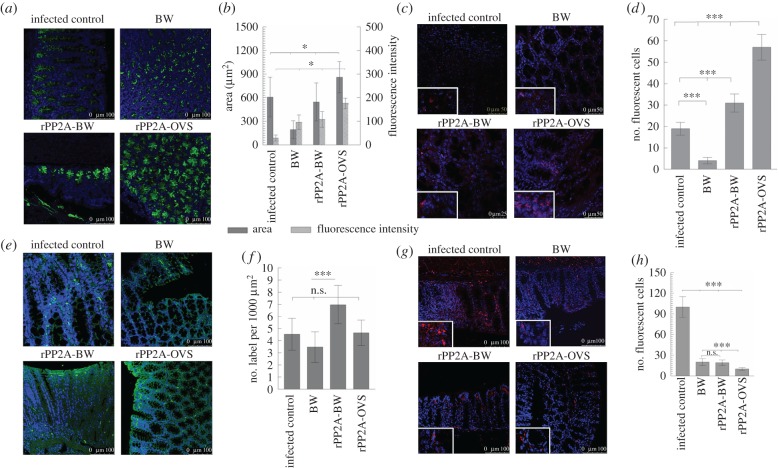
The evaluation in thin sections of the intestine of the immune response of the different groups by laser confocal microscopy. (*a*) Levels of fluorescent areas of the mucus of the crypt in the colonic mucosa, using WGA lectin labelled with FITC. (*b*) Quantification of the mean values of fluorescence in the different treated groups, lectin labelled. Dark bars correspond to the means area measured plus s.d. of the values; grey bars correspond to the means of fluorescence intensity plus s.d. of the values. (*c*) CCL11 (red labelled with Alexa-Fluor 633) in the thin section of the intestinally treated mice. (*d*) Means of the number of fluorescent marks to CCL11 plus s.d. of the mean values of cells measured in an area of 125 µm^2^. (*e*) OX40 (green labelled with FITC) in the thin section of the intestinally treated mice. (*f*) Average values plus s.d. of fluorescent cells of OX40 in an area of 1000 µm^2^. (*g*) Confocal microscopy studies of plasma cells (CD138 in red with Alexa-Fluor 633) in thin intestinal sections. (*h*) Average number and s.d. of plasma cells measured in an area of 170 µm^2^. The nuclei were labelled with DAPI (blue). BW corresponds to control animals immunized with bacterial walls; rPP2A-BW corresponds to the animal group immunized with the recombinant peptide plus bacterial walls and rPP2A-OVS is the group immunized with the self-assembling lipopeptide. Tukey–Kramer test, *p* *<* 0.001 (***), *p* *<* 0.01 (**) and *p* *<* 0.05 (*).

Analysis by confocal microscopy of the intestinal sections in different groups of mice was performed in order to evaluate the levels of mucus of the crypt in the colonic mucosa, using WGA lectin labelled with fluorescein. The results showed that the fluorescent areas inside the crypts were larger in animals treated with rPP2A-OVS than in the rest of the groups, and the intensity of the fluorescence was also stronger. These differences proved significant (Tukey–Kramer test, *p* < 0.001) compared with the other groups. On the contrary, the values of the area in the IC group and in the group immunized with rPP2A-BW proved similar (figure 4).

**Figure 5.**
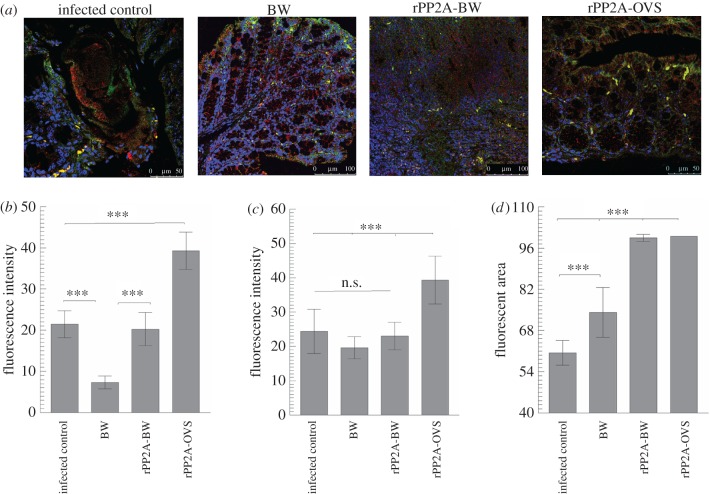
Confocal microscopy studies on neutrophil cells and CCL20. (*a*) Confocal microscopy of recruitment of neutrophils (green with FITC) and CCL20 (red with Alexa-Fluor 647) in thin intestinal sections in IC and mice immunized with BW, rPP2A-BW and rPP2A-OVS. The nuclei were labelled with DAPI (blue). (*b*) Means plus s.d. of the fluorescence intensity values of neutrophils measured in an area of 2000 µm^2^. (*c*) Means plus s.d. of the fluorescence intensity values of CCL20 measured in the intestinal crypts. (*d*) Percentage of fluorescent area plus s.d. of the values of CCL20 labels measured in the intestinal crypts. Tukey–Kramer test, *p* *<* 0.001 (***), *p* *<* 0.01 (**) and *p* *<* 0.05 (*).

Figure 5 presents both the confocal microscopic images of the recognition levels of CCL20 (Alexa-Fluor 647) and the recognition by the specific antibody with the fluorochrome fluorescein isothiocyanate FITC against the neutrophils in an area of 2000 µm^2^ in the sections of intestine from animals of the different groups, taking into account the fluorescence intensity values and the fluorescence percentage occupied with respect to the total area measured. It was found that when the sections were treated with the antibody against the neutrophils, the number of fluorescent cells appearing in the mucosa surrounding the crypts and on the intestine borders was larger, whereas a green colour was appreciable along the borders of the mucosa, likely to be a consequence of the secretion products of these neutrophils. In the IC, BW and rPP2A-BW groups, fluorescence intensity did not show significant differences; by contrast, the animals treated with rPP2A-OVS registered highly significant differences (*p* < 0.001) with respect to the other groups (figure 5).

The levels of recognition of CCL20, evaluated as the intensity of the red colour in the rPP2A-OVS group, showed significant intensity levels of fluorescence (*p* < 0.001, ***) in comparison with the other groups, while the IC and rPP2A-BW groups were significantly increased with respect to the BW group (figure 5). On the contrary, when the percentage of fluorescence was evaluated per surface area of the crypts, values proved significantly higher (*p* < 0.001, ***) in the groups that included the peptide rPP2A (rPP2A-OVS and rPP2-BW) as opposed to the IC and BW groups, although the percentage of fluorescence per unit of surface area in the BW group was significant compared to that shown by IC (figure 5).

The levels of fluorescence found when studying eotaxin (CCL11) (Alexa-Fluor 633) showed (figure 4) that the fluorescence appeared on the epithelium surrounding the crypts and the groups significantly differed from each other, especially the one treated with the lipopeptide and rPP2A, which registered the highest significance (*p* < 0.001, ***) in the intensity of fluorescence compared with the rest of the groups (figure 4).

The fluorescence levels for OX40 (FITC) are presented in figure 4*.* The signal appears preferentially on the brush border of the mucosa and in the epithelium that borders the crypts, and the intestines of the group rPP2A-BW had the highest expression levels, being non-significant in the other three groups (figure 4).

**Figure 6.**
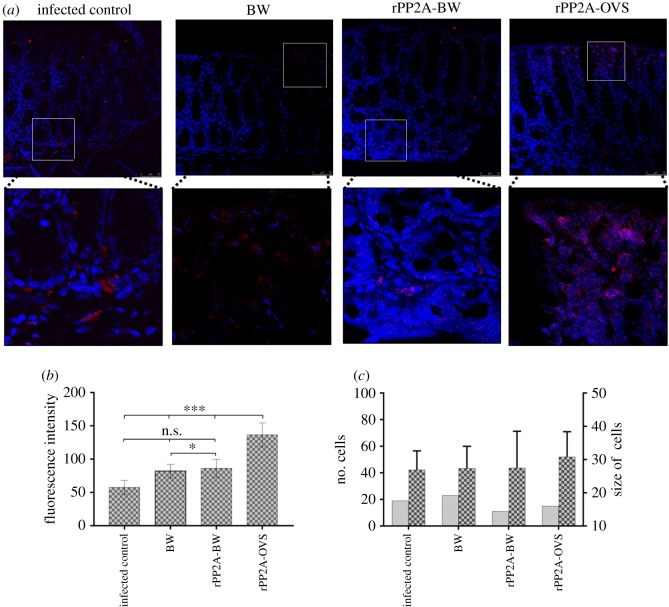
Confocal microscopy studies of Tuft cells. (*a*) Confocal microscopy studies of Tuft cells with anti-Dcamkl1 (red) in gut sections. (*b*) Means plus s.d. of the fluorescence intensity values of Tuft cells measured in an area of 138 µm^2^. (*c*) Number of cells (grey bars) and means plus s.d. of Tuft cells size (patterned bars). BW corresponds to control animal immunized with bacterial walls (BW); rPP2A-BW corresponds to the animal group immunized with the recombinant peptide plus bacterial walls, rPP2A-OVS is the group immunized with the self-assembling lipopeptide. Tukey–Kramer test, *p* *<* 0.001 (***), *p* *<* 0.01 (**) and *p* *<* 0.05 (*). The nuclei were labelled with DAPI (blue).

CD138 expression was highly specific for the plasma cells, given that this receptor was not expressed in the undifferentiated plasmablasts [27]. Figure 4 shows the location of the plasma cells CD138, near the lamina propria and inside the intestinal villi of the immunized mice. This marker indicated that the IC group significantly differed (*p* < 0.001) in terms of the number of cells with respect to the other groups, but no differences were detected among the immunized groups (figure 4). Regarding the count, the study of fluorescence intensity and size of Tuft cells using an antibody against Dcamkl1 and revealed with Alexa-Fluor 647 (figure 6) showed that the number of cells per area studied (a square of 138 µm per side) did not vary between groups, nor did the average size of the cells recognized by the antibody (figure 6). However, the fluorescence intensity per cell was highly significant (*p* < 0.001) in the rPP2A-OVS group in comparison with the other groups. The significance of the rPP2A-BW and BW groups showed significant values with respect to the IC group.

**Figure 7.**
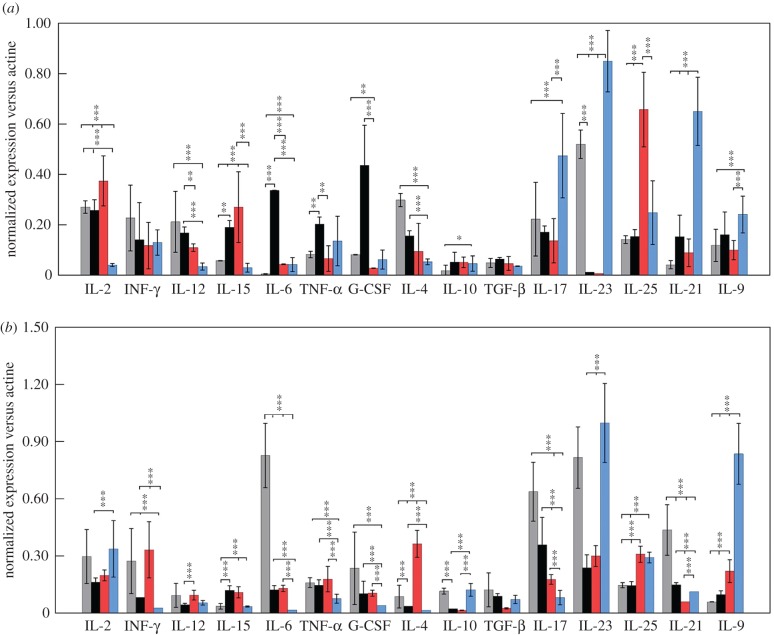
Determination by quantitative real-time PCR of the interleukin levels in the different immunized animal groups. (*a*) Study in mesenteric lymphatic nodules (MLN). (*b*) Study in Peyer's patches (PPs). The values are the means of the normalized expression values plus s.d. of these values. Tukey–Kramer test, *p* *<* 0.001 (***), *p* *<* 0.01 (**) and *p* *<* 0.05 (*). Grey bars, IC; black bars, animal groups immunized with bacterial walls (BW); red bars, animals immunized with rPP2A plus bacterial walls (rPP2A-BW); blue bars, animals immunized with the lipopeptide (rPP2A-OVS).

The analysis of the expression levels of the different interleukins was also studied by quantitative real-time PCR. In MLN (figure 7), a high IL-23 response was found, followed by IL-4 and IL-2, with a balanced value of IL-17 in the IC mice. Meanwhile, in the BW-intranasally immunized animals, there was an increase of G-CSF, IL-6 and IL-2 when compared with the other groups. However, in the groups immunized with rPP2A-BW, the predominant response was of IL-25 and IL-2. Also, in those immunized with rPP2A-OVS, a rise of IL-23 accompanied by IL-17, IL-9 and TNF-α was encountered.

In the PPs (figure 7) of the animals from the IC group, a high IL-23 response was developed, followed (in order of expression) by IL-6, IL-17 and IL-21, with relatively moderate levels of IL-2, IFN-γ and G-CSF. The BW group developed a high IL-17 response accompanied by a medium level for IL-23, though this proved 3.5-fold lower than that registered in the control group. The Th2 responses such as IL-4 and IL-10 were in general very low with the exception of the rPP2A-BW group, in which the maximum response was recorded for IL-4. In addition, this group registered the maximum response for IFN-γ and intermediate for IL-23 and IL-25, followed by IL-9. However, in the rPP2A-OVS immunized mice, the response in the PPs was mainly IL-23 and IL-9, followed by IL-25, with low levels for IL-17.

## 3. Discussion

The results found in this study clearly demonstrate that a recombinant version of the catalytic subunit of the enzyme serine/threronine phosphatase 2A (PP2A) from the nematode *A. costaricencis* formulated as a lipopeptide conjugated with OVS can induce significant cross-protection against *T. muris* infection in mice. This confirms the high immunogenicity of the PP2A where the immunoprotective capability of the recombinant peptide was corroborated for *A. costaricensis* [28] as well as other nematodes such as *Haemonchus contortus* and *Teladorsagia circumcincta* [9]*.* The results presented reinforce the notion that conjugated lipopeptides are excellent adjuvants. Although the underlying molecular mechanism that triggers these compounds is not fully understood, it is attributed to the capability of aggregating to form micelles or particles, protecting the epitope from degradation by serum enzymes [29–31] or, perhaps, determining the type of antigenic presentation, among other possibilities. In an aqueous medium, the hydrophobic parts of lipids would remain inside, forming a lipophilic nucleus, while the more hydrophilic peptides would stay outside, thus permitting greater efficiency in the presentation of the APC [32,33]. The OVS, without coupling to a protein, would bond by the high reactivity of the sulfone group to the amine groups of the proteins at the administration site, lacking activity as an adjuvant, as was determined in previous unpublished assays.

The safety of the different antigen preparations administered in the assay is evident as no weight changes in the inoculated animals were observed. In terms of the effectiveness of the immunological activation against *T. muris,* the number of nematode eggs present in the faeces abruptly decreased from the seventh day after intranasal administration of the preparations containing the lipopeptide (rPP2A-OVS) or rPP2A plus the BW (rPP2A-BW). It was striking that the group treated only with BW and without the recombinant antigen had a lower average number of eggs in faeces than did control, although not as markedly as with the peptide formulae. Other authors have previously reported the positive effect of both LPS and BW as protectors against helminth infection [34–36]. These effects may be attributed to the immunological activation by the LPS present in the BW that act as TLR-agonists in a wide range of cell types [37,38]. After their activation, the TLRs modulate the immune response, inducing the expression of the chemokine CCL20 [39,40]. Intestinal epithelial cells, among others, express this chemokine in case of inflammation [41–43]. The evaluation of the sections of the intestine in the different groups of mice by immunohistochemistry revealed that those treated either with rPP2A-OVS or with rPP2A-BW reached high levels of fluorescence in the area studied, as well as a significant number of cells that expressed CCL20, compared with the IC and BW groups. The level of fluorescence for this chemokine in the IC group could be a consequence of the ability of *T. muris* capable to stimulate TLR4 [44].

Although *T. muris* is a parasite specific to mice, not all inbred strains of mice are susceptible [45]. After the ingestion of the embryonated eggs of the nematode, even at very low doses, acute colitis develops in all the mice strains. However, parasitism results only in the mice in which an inflammatory response was triggered, while in the hosts that developed a Th2 response (e.g. in BALB/c mice), worms were expelled approximately 20 days post-infestation [46]. In AKR/OlaHsd mice used in these experiments, infestation triggered a Th1-dependent inflammatory response in the PPs, and therefore a chronic colitis developed, indicative of the persistence of the adult worms in the large intestine due to the inability of the host to expel them 20 days post-infestation [47–49]. In this study, the IC mice developed high levels of inflammatory Th17-dependent interleukin expression, IL-17, IL-23 and IL-21, as well as the expression of IL-6 in PPs. However, the expression levels of IFN-γ, IL-2 and GM-CSF, though not reaching expression values of IL-6, appeared high. Nevertheless, in the MLN, a clear Th1 increase was found together with a rise in IL-2, IL-12 and IFN-γ, with an increase in IL-4 (Th2) but not IL-10 (Treg), and a less notable rise in some of the Th17 interleukins such as IL-23 and IL-17. This leads to the assumption that the Th17 inflammatory response in the PPs corresponds to a local type of Th17 response, while the one corresponding to the MLN would be a mixed Th1/Th17 systemic response, perhaps mediated by the expression of CCL20 in the intestinal epithelium cells.

The first line of defence encountered by nematodes in their intestinal habitat is the mucosal barrier. The surfaces of the intestinal cells are coated by a gel that constitutes a mucus made up primarily of mucins, a series of glycoproteins that are secreted by the goblet epithelial cells, this constituting an essential component in the defence and elimination of gastrointestinal helminths [50–53]. Mucins are the major component of the mucus secreted by the goblet cells, but other molecules such as antibodies, defensins and lysozymes are also present, covering the entire intestinal epithelium [54,55]. The layer of mucus keeps the intestinal surface covered with peptides that show a bactericide action ensuring its sterility, and also have an activity against infection by parasites, including nematodes, due to the presence of different bioactive factors [56,57]. Furthermore, the physical barrier of the mucus may also interfere in both the feeding mechanisms as well as the mobility of the worms. This could explain why hyperplasia of the goblet cells occurs in nematode infections and the hyperplasia that might be induced by IL-9 [50,58]. In our results, the highest levels of fluorescence intensity and area occupied by the mucus were found in the groups treated with lipopeptide (rPP2A-OVS) and rPP2A-BW in which worm reduction was most successful. The analysis of mucus production by WGA lectin demonstrated that these two formulae administered intranasally are capable of stimulating, by MALT, the mucus-producing goblet cells of the intestinal mucosa. As evidenced with the levels of eggs in faeces, the percentages of reduction of parasitism in the different groups ranged from 24.47% (BW treatment) to 98% (lipopeptide). The reduction after BW administration was lower than that observed for rPP2A-BW (59%), demonstrating the effectiveness of the stimulation of the response by the recombinant antigen. Also, when these BW were administered together with the recombinant antigen (rPP2A) the percentage of reduction in the number of worms would be comparable with levels found in other models of parasitic gastrointestinal nematodes of livestock when the same rPP2A-BW combination is used [59]. On the other hand, when rPP2A was linked with oleic-vinyl sulfone (rPP2A-OVS), the reduction rates of parasitism reached their highest levels, practically 98%. This group of animals registered the maximum fluorescence levels when glycoprotein production in the mucus was analysed and showed the highest expression levels of the chemokines CCL20 and CCL11, both linked to the process of cell attraction to the inflammation site and stimulation of goblet cells. In addition, it is known that the Th17 cells recruit neutrophils [60], and this could explain not only the increase in neutrophils that appear in the mice of the rPP2A-OVS group but also the high IL-17 expression levels.

In addition to the goblet cells, other cells such as M-cells [61], Cup-cells [62] and Tuft cells [63] in the intestinal epithelium also regulate the immune response of the mucosa. Until very recently, the function of these cells was unknown, but they have been described as participating very actively in the immunological regulation against parasites. Tuft cells are differentiated from the rest of the mucosal cells for having a tubule-vesicular system and a plume of villi towards the luminal side, giving them the name ‘Tuft’. In this group of tubules, microtubule-linked protein kinase Dclk1 is found, known as Dcamkl-1 [64], that allows these cells to be recognized by antibodies against this kinase. It is known that the number of Tuft cells increases with the presence of parasites [65–67]. In parasite-free animals, the number of Tuft cells of the intestinal epithelium ranges from 0.4 to 1% of the total of the epithelium cells [64]. In our experiments, the number of cells stained with anti-Dcamkl-1 did not vary among groups and neither did their size. A possible explanation is that in all the groups the infections occurred with a similar number of nematode larvae, and thus the parasitism undoubtedly boosted the number of Tuft cells, which in our case ranged between 13 and 20 in the study area (a square of 138 µm per side) for each of the different treatments. Despite the reduction in the number of worms found in the rPP2A groups, the parasite was not totally eradicated in any group. Consequently, we conclude that the decline in the number of Tuft cells could not have taken place without the absence of parasites in the intestinal mucosa [65]. However, the level of fluorescence, as indicative of the activity of the Tuft cells in the IC group, was significantly lower than in the other three groups, especially in comparison with the groups treated intranasally with the lipopeptide. This could indicate that the presence of the antigens, together with the BW or linked to the OVS, stimulated the activity of these cells.

Intestinal bacteria also reportedly promote IL-25 (IL-17E) production through Tuft cells [68]. A similar response may involve the nasal mucosa, implying a stimulation of the Tuft cells mediated by the cooperation between the NALT-GALT systems by some of the stimulation factors such as chemokines. This could account for the high expression levels of IL-25 in the MLN of the mice inoculated with the preparations that carried the recombinant antigen (rPP2A-BW and rPP2A-OVS). Owyang *et al*. [69] found that IL-25 is expressed both in the caecum as well as in the MLN, and the animals resistant to *T. muris* infection present significantly higher levels in the caecum than in the MLN. The mice strain AKR/OlaHsd, susceptible to infections and used in this study, registered comparable IL-25 expression levels both in the MLN and the caecum. Tuft cells are currently considered the prime source of parasite-induced IL-25 production, and IL-25 promotes the production of IL-13 by innate lymphoid C2 (ILC2) cells.

In our study, the group of mice that were vaccinated with rPP2A-BW showed higher levels of IL-25 expression in the MLN versus the expression levels in PPs. The IL-25 expression level in MLN from the mice treated with rPP2A-OVS did not correspond to the levels of IL-25 in the PPs, where the values of this group were similar to those of rPP2A-BW, although both were significantly higher than in the two other control groups (IC and BW). However, as noted above, expression levels differed from those found in the MLN, whereas IL-9 expression levels proved far higher in the PPs than in the MLN. Curiously, the group treated with the lipopeptide registered the highest expression levels of this interleukin in the PPs.

It is known that T cells *in vitro* stimulated with TGF-β and IL-4 express IL-9 and produce high levels of mRNA for the IL-17RB receptor, the receptor for IL-25. In addition, the treatment of these T cells with IL-25 boosts the expression of IL-9, confirming the induction of this interleukin by IL-25 [70]. This might imply that the effect on the expulsion of the worms attributed to IL-25 could be potentially mediated by IL-9, able to stimulate the production of IL-13, as observed prior to the anti-parasite effect [71]. IL-9 is pleiotropic and, among other biological effects, reduces the expression of the claudin2 protein of the tight junction of the intestinal cells. Thus, IL-9 production by these cells could alter the function of the intestinal barrier [72], altering the intestinal flow towards the intestinal lumen [73]. Another role of this interleukin is the effect on intestinal contractibility, favouring the expulsion of the worms [74].

In summary, the results of the present study suggest that the treatment of the AKR/OlaHsd mice susceptible to infection by *T. muris*, by the nasal immunization with the recombinant peptide rPP2A as a lipopeptide bound to OVS and administered at the outset of the chronic phase of infection, is able to activate a combined Th17/Th9 response orchestrated by the cytokines IL-25, IL-17 and IL-9, restraining egg release by intestinal worms and resulting in accelerated worm expulsion. This strategy of immunization could be of great applicability not only in immunotherapy and immunoprophylaxis to control diseases caused by nematode parasites of the intestinal mucosa but also in those pathologies in which it is necessary to act at the level of the Th9 response in the mucosa.

## 4. Material and methods

### 4.1. Antigen

We used a recombinant peptide expressed by a CT2–2 clone corresponding to the PP2A catalytic region of *A. costaricensis* [28] as the antigen. Recombinant production was previously described by Fawzi *et al*. [9] and briefly described below. The protein expression in the recombinant bacteria was induced with 0.5 mM IPTG (isopropyl-β-d-thiogalactopyranoside). The recombinant protein was purified by affinity chromatography with HisTrap FF Crude column (GE Healthcare Life Sciences, 11-0004-58), previously equilibrated with binding buffer (6 M guanidine-HCl, 20 mM sodium phosphate, 500 mM NaCl, 5 mM imidazole, 1 mM β-mercaptoethanol, pH 7.4). The sample was loaded, and the column was washed with binding buffer and a gradient of imidazole from 20 to 60 mM. The recombinant protein was eluted with elution buffer (6 M guanidine-HCl, 20 mM sodium phosphate, 500 mM NaCl, 500 mM imidazole, 1 mM β-mercaptoethanol, pH 7.4). After dialysis, the protein concentration was determined by the Bradford method in denaturizing buffer (6 M guanidine-HCl).

### 4.2. SDS-PAGE electrophoresis and western-blot analysis

A defined amount of 20 µg per lane of protein was subjected to 12.5% SDS-PAGE and stained with Coomassie Brilliant Blue. Unstained replicas were transferred to 0.2 µm PVDF membranes (Bio-Rad, 170-4156) using a Bio-Rad Trans-Blot Turbo TM (0.6 Å, 25 V, 40 min). Western blot analyses were carried out using sera obtained from mice immunized with PP2A [9] and diluted 1 : 150 with PBS-T (0.3% tween-20 in PBS). Membranes were washed with phosphate-buffered saline with 0.05% Tween 20 (PBS-T), incubated for 2 h at 37°C with 1 : 1000 polyclonal Goat anti-Mouse Immunoglobulins/HRP (Dako Cytomation-P0447) and developed using chemiluminescence (ClarityTM Western ECL Substrate, Bio-Rad, 170-5060).

The protein band from the SDS-PAGE electrophoresis recognized by the sera was sequenced by fingerprinting and identified at the Servicio de Proteómica del Centro de Biología Molecular Severo Ochoa (CBMSO) in Madrid (Spain) in an Autoflex matrix-assisted laser-desorption ionization time-of-flight (MALDI-TOF) mass spectrometer (Bruker) equipped with a reflector following a previously described protocol [75].

### 4.3. Synthesis of alkyl vinyl sulfone

The synthesis of (9Z)-*N*-[2-(ethenylsulfonyl)ethoxy]-ethyl]-9-octadecenamide (OVS) has been previously described in detail [76] and is summarized in figure 1.

### 4.4. Coupling of the alkyl vinyl sulfone to rPP2A and mass determination

The synthesized alkyl vinyl sulfone was solubilized in methanol and mixed with 0.125 mM carbonate buffer (pH 8.3) to a final concentration of 2 mg ml^−1^. The solution of the alkyl vinyl sulfone was mixed with the purified rPP2A (2 mg ml^−1^) in carbonate buffer (0.125 mM carbonate, pH 8.3) and kept at 4°C in an orbital shaker for 12 h. Thereafter, free reactive groups were blocked with a molar excess of glycine in carbonate buffer at room temperature for 4 h. The conjugates were dialysed for 12 h against a solution of ammonium acetate (0.1 M, pH 7) and then lyophilized.

Mass spectrometer analyses were performed at Laboratorio de Espectrometría de Masas (SIdI), Faculty of Sciences, UAM, Madrid (Spain) in a Ultraflex III TOF/TOF instrument (Bruker) that uses an NdYAG laser (emission, 355 nm; accelerating voltage, 25 kV). The sample (0.5 mg of lipopeptide in 0.2 ml of 1,1,1,3,3,3 hexafluoruro-2-propanol) and matrix solution (10 mg of α-ciano-4-hydroxycinnamic acid (ACC) in 1 ml of acetonitrile, 0.1% TFA in water 3 : 1 v/v), were mixed in a proportion 1 : 20, and then applied to a metal sample plate for MS analysis. The lipopeptide mass was measured at approximately 1000 Da absorption laser intensity.

### 4.5. Electron microscopy

Electron microscopy was used to check whether the alkyl functionalized peptides were aggregated in micelles and to estimate their size. Hence, the lipopeptide was sonicated in a 0.1 M ammonium acetate solution and visualized in a transmission electron microscope (TEM) (Libra 120 PLUS de Carl Zeiss SMT) by depositing 5 µl of the suspension adsorbed directly onto a 300 mesh Cu grid covered with Formvar and stained with 1% uranyl acetate for 1 min [77]. To determine the size of the micelles, 100 of the observed particles were measured digitally using ImageJ software (v. 1.48 e, Wayne Rasband, htpp:\\imagej\nhi\gov\imagej).

### 4.6. Animal experiments

The inbred AKR/OlaHsd mouse strain was used in all experiments. This mouse strain is highly susceptible to *T. muris* infection and, following a primary infection, it allows the progression to a chronic stage with persistence of fecund adult worms due to activation of an inappropriate Th1-type response [78].

A total of 36 inbred female AKR/OlaHsd, aged six to eight weeks, were purchased from Harlan Laboratories UK Ltd (Blackthorn, Bicester, UK) and maintained under specific pathogen-free conditions at the VISAVET animal house facilities at Complutense University of Madrid with ad libitum access to food and water, regulated temperature and light/dark cycle conditions. For the experiments, the mice were reared in six randomly assigned groups and placed in the corresponding standard methacrylate cages for the duration of the experiments. All mice were weighed weekly along the time of the experiments.

### 4.7. Parasites

The Edinburgh strain of *T. muris* (isolated in 1961 [79] and since then maintained by periodical passage in outbred mice) was used in this study. The methods for the maintenance and infection were the same as previously described [80]. Each mouse was infected with approximately 200 embryonated eggs by oral gavage on day 0.

### 4.8. Assessment of infection and immune response

For an assessment of the level of infection as well as the local and systemic immune response elicited against infection, the mice were killed by an overdose with isoflurane, placed under aseptic conditions, and their large intestines removed and longitudinally opened. Intestinal L-3 larvae were released and collected as previously described [80], and then counted under a stereomicroscope.

MLN and PPs samples were also taken, and placed in RNAlater buffer for further cytokine measurement. Likewise, the ending tip of the caecum from each animal was removed and placed in a solution of 0.5% glutaraldehyde and 2.5% paraformaldehyde buffered at pH 7.4 for further immunohistochemical studies.

### 4.9. Immunization schedule and follow-up

In this study, the immunization schedule was designed on the basis of the demonstrated susceptible phenotype of the AKR/OlaHsd mouse strain regarding *T. muris* infection, thus allowing the establishment of a chronic infection as reported above [78]. Our rationale was to check whether the predominant Th1 response elicited during the first three weeks of infection leading to susceptibility could be reversed to resistance by applying a recombinant vaccine throughout the chronic stage of the infection able to trigger a predominant Th2 response and thus leading to expulsion of the established mature worms.

For this, on day 49 p.i. the animals received the first immunization dose. Three freeze-dried vaccines—bacterial walls (BW) and free rPP2A protein plus (BW) at equivalent amounts to those given separately to the corresponding groups and rPP2A coupled to a lipid OVS—were suspended in 1% carboxymethylcellulose to reach a concentration of 0.8 µg ml^−1^. Four micrograms of each preparation were administered to mice by nasal instillation of 5 µl of the antigenic suspension applied into the right nostril using a micropipette coupled to a special fine long tip. An additional infected control (IC) group was administered with PBS (0.25 M plus the 1% carboxymethylcellulose). Boosters were applied two weeks later to all groups in the same way as the first administration vaccine dose.

To monitor the effectiveness of the vaccine, faecal egg counts were performed weekly by the Stoll method, from the day of the first vaccination until the day of killing the mice. Briefly, the faeces released by each animal temporary isolated for 8 h in an individual cage were collected and homogenized in 0.1% NaOH (4 g/60 ml 0.1 N NaOH rate). Six aliquot fractions of the suspended material were placed on glass slide, and eggs were counted under an optical microscope at 4× magnification. The results were expressed as the number of eggs/gram of faecal samples. Sixty-three days after the first vaccination all animals were killed and processed for adult intestinal-worm collection and counting, as well as for immunochemical studies.

### 4.10. Isolation of mRNAs, synthesis of cDNAs and quantitative real-time PCR

Total RNA was isolated from MLN and PPs preserved in RNAlater, an RNA stabilization reagent (Qiagen-76104) using the RNeasy Midi kit (Qiagen-74106). Once the RNA was isolated, the sample was digested with RNase-Free DNase Set (Qiagen-79254), in order to remove DNA contamination. For mRNA isolation, the Oligotex mRNA Midi kit (Qiagen-70042) was used. The quality of the purified mRNA was measured in an Experion automated electrophoresis system (Bio-Rad, Nazareth Eke, Belgium). Subsequently, cDNA was generated with the iScript cDNA Synthesis kit (Bio-Rad, 170-8891) in a CFX96 real-time system (Bio-Rad). Thus, a 20 µL final volume reaction containing 4 µl of 5× iScript reaction mix, 1 µl de iScript reverse transcriptase, and an mRNA volume between 100 fg and 1 µg, was performed. The retrotranscription conditions were 5 min at 25°C, 30 min at 42°C, 5 min at 85°C at the end the sampling, and conditions were maintained at 12°C. The concentration and quality of the cDNA was calculated spectrophotometrically in a Nanodrop (Nanodrop ND-1000, Thermo Scientific). The cDNA was diluted 1 : 10 and preserved at −80°C until use.

Quantitative real-time PCR was performed on a thermocycler (CFX96 Real-Time System, Bio-Rad) in combination with primers specifically designated using the eprimer3 software (http://www.bioinformatics.nl/cgi-bin/emboss/eprimer3) and shown in electronic supplementary material, table S1. The cDNA was quantified using SsofastTM Eva Green Supermix (Bio-Rad, 172-5201), with cDNA equivalent to 50 ng mRNA. The cycling conditions were as follows: 95°C for 2 min, followed by 40 cycles of 95°C for 10 s, 55°C for 30 s and 60°C for 30 s. When the amplification was completed the samples were kept at 12°C. The primer concentration was optimized and dissociation curves were generated for each primer set to verify the amplification of a single PCR product. Expression of β-actine RNA [81–83] was used to normalize the expression of other genes quantified according to the ΔCT method, in which the β- actin: Interleukin ratio = 2 CT β-actin − CT interleukin. All of the assays were done in triplicate.

### 4.11. Immunohistochemistry of mouse tissues

For histological evaluation, the glutaldehyde/paraformaldehyde-fixed tissues were thawed in PBS at laboratory temperature under permanent automatic rotation of the sample tube. Then, the samples were trimmed and embedded in paraffin wax. Sections of 8–10 µm were affixed to slides. The paraffin was removed by three dips of 15 min in xylene and an alcohol series (100%, 90%, 70%), and one rinse in water [84]. After paraffin removal and hydration, the slides were prepared for antigen retrieval by heating at 120°C for 10 min in an autoclave with 0.01 M citric acid at pH 6.0 [85,86].

After the unmasking treatment of the antigens, as a means to prevent the non-specific binding of the antibodies, a treatment was made for 30 min with PBS containing 1% of albumin from chicken egg white (Sigma, A5503). The slides were treated with different specific antibodies, as shown in electronic supplementary material, table S2, at a concentration of 1 : 50 for 1 h at room temperature. Finally, cell nuclei were stained with a dilution 1 : 100 of 4′,6-Diamidino-2-phenylindole dihydrochloride (DAPI) (Sigma, D9542) for 10 min. They were subsequently preserved and mounted in a mounting medium (Prolong Antifade Kit, Molecular Probes) and examined under a Leica DMI6000 confocal laser microscope equipped with a filter system for FITC (mean wavelength 530 nm, maximum 490 nm). Electronic supplementary material, table S2 shows the origin and labelled antibodies and reagents for the immunocytochemistry by confocal studies.

### 4.12. Statistical analysis

The Tukey–Kramer multiple-comparisons test was used to estimate the significance of the difference between means. The results are expressed as mean values (standard deviation of the different groups at different times for each experiment performed). *p* < 0.001 was considered to be highly significant (***), *p* < 0.01 was considered moderately significant (**) and *p* < 0.05 was considered a low level of significance (*). GraphPad Instat v. 3.05 software (GraphPad Software, Inc., La Jolla, CA, USA) was used for the statistical analysis.

## Supplementary Material

Table S1 from Self-adjuvanting C18 Lipid Vinylsulfone-PP2A vaccine: study of the induced immunomodulation against Trichuris muris infection by M. Gomez-Samblas, JJ. García-Rodríguez, M Trelis, D. Bernal, FJ Lopez-Jaramillo, F Santoyo-Gonzalez, S. Vilchez, AM Espino, F. Bolás-Fernández, A. Osuna; Table S2 from Self-adjuvanting C18 Lipid Vinylsulfone-PP2A vaccine: study of the induced immunomodulation against Trichuris muris infection by M. Gomez-Samblas, JJ. García-Rodríguez, M Trelis, D. Bernal, FJ Lopez-Jaramillo, F Santoyo-Gonzalez, S. Vilchez, AM Espino, F. Bolás-Fernández, A. Osuna

## Data accessibility

The datasets supporting this article have been uploaded as part of the electronic supplementary material.
