# Supplementary material for: Self-adjuvanting C18 lipid vinil sulfone-PP2A vaccine: study of the induced immunomodulation against Trichuris muris infection
Source: Open Biol. 2017 Apr 12;7(4):170031. doi: 10.1098/rsob.170031 (PMC5413912; doi:10.1098/rsob.170031)
Supplement: Table S1 from Self-adjuvanting C18 Lipid Vinylsulfone-PP2A vaccine: study of the induced immunomodulation against Trichuris muris infection by M. Gomez-Samblas, JJ. García-Rodríguez, M Trelis, D. Bernal, FJ Lopez-Jaramillo, F Santoyo-Gonzalez, S. Vilchez, AM Espino, F. Bolás-Fernández, A. Osuna; Tab [file rsob170031supp1.pdf]

Table S1. Primers sequence of interleukin genes used in this study

Primer	Sequence (5` - 3`)	
Act	Sense	TCCATCATGAAGTGTGACGT
	Antisense	GAGCAATGATCTTGATCTTCAT
G-CSF	Sense	CAGAGGCGCATGAAGCTAAT
	Antisense	TCCAGGGACTTAAGCAGGAA
IL2	Sense	CCACTTCAAGCTCCACTTCA
	Antisense	ATCCTGGGGAGTTTCAGGTT
IL4	Sense	CCTCACAGCAACGAAGAACA
	Antisense	ATCGAAAAGCCCGAAAGAGT
IL6	Sense	AGTTGCCTTCTTGGGACTGA
	Antisense	TCCACGATTTCCCAGAGAAC
IL9	Sense	TGATTGTACCACACCGTGCT
	Antisense	AGGTCACTCCAACGATACGG
IL10	Sense	CAGAGCCACATGCTCCTAGA
	Antisense	TCATTTCCGATAAGGCTTGG
IL12	Sense	GACCAAACCAGCACATTGAA
	Antisense	CTACCAAGGCACAGGGTCAT
IL15	Sense	CATTTTGGGCTGTGTCAGTG
	Antisense	TGCAACTGGGATGAAAGTCA
IL17	Sense	TCCAGAAGGCCCTCAGACTA
	Antisense	TCATGTGGTGGTCCAGCTT
IL21	Sense	GAGGACCCTTGTCTGTCTGG
	Antisense	TCATCTTTTGAAGAAGCCATTT
IL23	Sense	TAATGTGCCCCGTATCCAGT
	Antisense	AGGCTCCCCTTTGAAGATGT
IL25	Sense	CGGAGGAGTGGCTGAAGTGGAG
	Antisense	ATGGGTACCTTCCTCGCCATG
INF-γ	Sense	GCTCTTCCTCATGGCTGTTT
	Antisense	GTCACCATCCTTTTGCCAGT
TNF-α	Sense	CCCCAAAGGGATGAGAAGTT
	Antisense	CACTTGGTGGTTTGCTACGA
TGF-β	Sense	TGGAGCAACATGTGGAACTC
	Antisense	AGCCCTGTATTCCGTCTCCT

Table S1. Labelled antibodies and lectin

**Primary antibody**	Fluorochrome	Cat. Nº	**Secondary antibody**	Fluorochrome	Cat. Nº
anti-CD134 / OX40 [OX-86]	FITC green	ab33998			
rabbit anti-CCL20/MIP3 alpha		Bs-1268R	Goat anti-Rabbit IgG (H+L)	Alexa Fluor® 647	A-21247
Anti-Neutrophil [7/4]	FITC	ab53453			
Mouse CCL11/Eotaxin Antibody		MAB420	Goat anti-Rat IgG (H+L)	Alexa Fluor® 633	A21094
Anti-DCAMKL1 antibody		Ab31704	Goat anti-Rabbit IgG (H+L)	Alexa Fluor® 647	A21245
Anti-mouse CD138		142502	Goat anti-Rat IgG (H+L)	Alexa Fluor® 633	A21094
**Lectin**					
Lectin from *Triticum vulgaris* (WGA)	FITC	L4895			
